# Examining the protective effect of motherhood status on alcohol use: comparing outcomes of US Army Reserve and National Guard Soldiers and Civilian Women

**DOI:** 10.1007/s10865-026-00679-x

**Published:** 2026-05-29

**Authors:** Malaiikha N. McCormick-Cisse, D. Lynn Homish, Gregory G. Homish

**Affiliations:** Department of Community Health and Health Behavior, University at Buffalo, Buffalo, NY USA

**Keywords:** Motherhood, Alcohol use, Military, USAR/NG Soldier, Mental health

## Abstract

Women are the fastest-growing demographic in the United States military and face increased risks for alcohol-related problems. Motherhood often protects against alcohol misuse in civilian populations; however, it is not known if the demands of multiple, competing intersecting roles of reserve soldiers can complicate this. We hypothesize that civilian mothers will endorse fewer alcohol problems than non-mothers, but that this protective effect will not extend to women in military roles. Data are drawn from women (N = 411) in Operation: SAFETY (Soldiers and Families Excelling Through the Years). The Alcohol Use Disorder Identification Test (AUDIT) was used to assess alcohol use (AUDIT Total, AUDIT ≥ 8, Consumption, Dependence, Alcohol-Related Harm), the Patient Health Questionnaire assessed depression, and the PTSD-Checklist for DSM-5 assessed PTSD. T-test and chi-square tests compared potential covariates. Logistic and negative binomial regressions examined alcohol use outcomes based on maternal status for civilian and military women separately. Adjusted models controlled for mothers’ age, education, depression, and PTSD symptoms. Motherhood status significantly influenced alcohol use outcomes. Compared to non-mothers, civilian mothers reported significantly fewer alcohol-related problems in all unadjusted and adjusted models (ps < 0.05). However, among women in USAR/NG roles, there was no significant difference in any alcohol use outcome by maternal status. Motherhood status was associated with lower risk alcohol use among civilian women. However, this protective relationship was not observed among women in USAR/NG roles. Subsequent research is needed to help better understand the alcohol use of military mothers.

## Introduction

Since the abolition of the all-male draft in 1973 and the removal of sex-based occupational restrictions in 2016, the number of women serving in the United States (US) military has steadily increased (Cazares et al., 2021). Women now represent one of the fastest-growing demographic groups among active-duty, reserve, and veteran populations (Adams et al., 2021; Mathes Winnicki et al., 2024). According to the 2023 US Department of Defense profile of the military community, women comprised approximately 21% of the United States Army Reserve and National Guard (USAR/NG) components (Defense, 2023). Although women only account for 10.5% of all veterans, projections suggest that they will account for 16–18% by 2040 (Adams et al., 2021; Goldstein et al., 2025). Growth is said to be particularly evident in USAR/NG components (Adams et al., 2021), where recent recruitment efforts have contributed to a surge in female enlistments (Beynon, 2025).

The inclusion of women in military roles has been associated with a more diverse workforce and enhanced mission performance (Cazares et al., 2021). However, servicewomen experience distinct physical, psychological, and social outcomes that can affect both individual and force well-being (Goldstein et al., 2025). Research suggests that female veterans are at an increased risk for psychological problems, potentially due to sex-specific exposures including military trauma, harassment, and gender isolation (Adams et al., 2021; Williamson et al., 2023). Compared to males, female veterans are also more likely to meet criteria for anxiety, post-traumatic stress disorder (PTSD), depression, and suicidal ideation, indicating greater vulnerability to complex multi-comorbidity following military service (Adams et al., 2021; Cazares et al., 2021; Goldstein et al., 2025).

These issues are particularly relevant within the USAR/NG components, which together comprise one-third of the total US military force (Defense, 2023). USAR/NG soldiers face unique stressors associated with part-time service, including frequent transitions between civilian and military responsibilities and environments (Hoopsick et al., 2018). Despite having similar occupational roles, compared to active duty military personnel, USAR/NG soldiers are at an increased risk for substance use and psychiatric problems, including suicidal ideation, PTSD, and alcohol use disorder (AUD) (Arif et al., 2024; Hoopsick et al., 2021, 2024). These issues may be particularly relevant for women in USAR/NG roles, who report similar lifetime substance use rates to their male counterparts (Hoopsick et al., 2022), but are more likely to report negative experiences such as military sexual trauma (Fillo et al., 2023).

Across US and international military populations, alcohol misuse remains a persistent issue (Osborne et al., 2022). Historically, alcohol has been used to mediate combat associated stress and to increase social bonding and camaraderie among soldiers (Jones & Fear, 2011; Osborne et al., 2022). Despite these perceived benefits of use, heavy and binge drinking have serious consequences for soldiers’ physical functioning, mental health, and overall operational readiness (Meadows et al., 2023; Osborne et al., 2022). National data indicate that 40.8% of veterans meet lifetime criteria for an AUD, with 10.5% meeting criteria within the past year (Panza et al., 2022). Among reserve members, 74% report past-month alcohol consumption (Hoopsick et al., 2018).

Women are particularly susceptible to the harmful effects of alcohol and experience a faster progression from initiation to dependence than men (Greaves et al., 2022; Kulak et al., 2021; White, 2020). Previous studies have reported that compared to men, women are more likely to use alcohol to cope with mental health issues, including PTSD and depression (Creech & Borsari, 2013; Fleming et al., 2025; Nunnink et al., 2010; Sairsingh et al., 2018). However, despite the slight increase in attention to alcohol use among civilian women (Grucza et al., 2018), research on female service members and veterans remains extremely limited and mixed (Goldstein et al., 2025; Mathes Winnicki et al., 2024; Osborne et al., 2022). Much of the existing literature has focused on general risk factors, while little is known about how social roles (e.g., motherhood) and conflicting responsibilities (e.g., military service) influence women’s drinking behaviors.

There are unique harms associated with drinking during certain life stages including pregnancy and motherhood (Fleming et al., 2025). While motherhood often serves as a protective factor against alcohol misuse in civilian populations (Balan et al., 2014), current research on alcohol use during motherhood is lacking likely due to social and cultural norms (Fleming et al., 2025). In addition, previous findings indicate that low mood (depression and stress) is associated with increased negative alcohol use behaviors among mothers (Fleming et al., 2025). Importantly, working mothers face many demands related to balancing work and family responsibilities which can lead to increased stress, limited resources, and conflicting role expectations (Patsouras et al., 2025). These factors can contribute to high-risk alcohol use behaviors of working mothers. Drinking patterns have also been found to vary by type of employment, suggesting that occupational context plays a meaningful role in influencing the alcohol use behaviors of women (Patsouras et al., 2025).

While every occupation has associated health risks, certain occupations come with greater physical and mental health demands than others (Adler & Castro, 2013). To better understand why military service in particular may lead to identity strain and role conflicts among mothers, the Occupational Mental Health Model can be used. The Occupational Mental Health Model is a framework for understanding the relationship between specific occupational demands and subsequent mental health adjustment, and goes beyond other models of this nature to include personal and occupational background variables (Adler & Castro, 2013). The model includes four key components: background, demand, outcome, and goal, as well as two moderating factors. Background factors include personal (trauma, or prior mental health history) or occupational (higher rank, or membership in elite units), demand encompasses personal, traditional occupational, and high-risk factors, outcomes include symptoms/reactions, attitudes, and performance, and lastly, goals can entail health, work, relationships and simply enjoying life (Adler & Castro, 2013). This model also accounts for moderation from personal and occupational resources (Adler & Castro, 2013).

Compared to other professions, military service and culture have distinct features that impact employee outcomes and goals (Adler & Castro, 2013). Combat, for example, is inherent within the military and demands of killing, avoiding being killed, caring for wounded individuals, and witnessing injury and death are all part of the job (Adler & Castro, 2013). Additionally, things like frequent relocation, separation from the family, and constant availability all add to the occupational experiences of military mothers. Military culture and beliefs, such as “mission first,” acknowledge that service members often have to sacrifice personal well-being or preferences to meet the requirements of a particular mission (Adler & Castro, 2013).

The USAR/NG may also produce a unique set of workplace stressors and environmental demands that alter alcohol outcomes. USAR/NG soldiers provide key support in both domestic operations (e.g., disaster response, managing politically charged events, public health missions, etc.) as well as overseas combat related deployments, exposing them to a wide array of high risk experiences (Cohen et al., 2015). Compared to their active duty counterparts, USAR/NG members often maintain “day jobs” and typically serve one weekend a month and 15 days annually (Cohen et al., 2015). Following deployment, USAR/NG soldiers also face unique readjustment challenges such as deploying without one’s unit, managing family and relationship difficulties, uncertain employment or rapid resumption of pre-employment civilian roles and expectations, and eligibility concerns surrounding health services that have been found to increase the risk for psychiatric disorders (Cohen et al., 2015). These experiences can lead to negative health behaviors such as substance use, and mental health outcomes such as PTSD, which may uniquely impact motherhood experiences of women in USAR/NG roles.

Despite the information gained from mental health-related findings from civilian populations, not all of the outcomes directly apply to certain occupational contexts, making this an important factor to consider (Adler & Castro, 2013). Currently, 34.5% of active-duty members have children (Defense, 2023). Although the transition to motherhood has been often considered protective in general populations, the long-term effect of motherhood on alcohol use remains poorly understood, specifically among military populations (Balan et al., 2014). Understanding alcohol use among women in the USAR/NG has important implications for soldier readiness, retention, and for the development of interventions that target the unique needs of servicewomen.

Much of the existing literature on military families has focused on fathers, their civilian spouses, and children; leaving the experiences of military mothers under-represented (Williamson et al., 2023). A growing body of work has begun to examine how factors such as perinatal mental health, PTSD, depression, and gender isolation influence military women’s health outcomes (Cazares et al., 2021). However, the risk and protective factors for AUD among servicewomen are understudied and warrant additional research, especially among women with multiple intersecting and overlapping roles such as women that are mothers (Mathes Winnicki et al., 2024; Straus et al., 2020). To our knowledge, no prior studies have evaluated how motherhood influences the alcohol use outcomes of USAR/NG women, a critical gap given the growing number of mothers in reserve and veteran populations that face complex caregiving and occupational demands.

The goal of the present study is to examine the impact of motherhood and military status on alcohol use outcomes among a sample of USAR/NG soldiers and civilian women. We hypothesize that civilian mothers will be less likely to endorse alcohol problems than non-mothers and that this protective effect of motherhood will not be observed among women in USAR/NG roles. Specifically, we aim to contribute to the literature on women who have ever served in the military by assessing whether having children is associated with a decreased risk of adverse alcohol outcomes and by determining if this relationship persists when considering covariates including the mothers age, level of education, and mental health symptoms (PTSD and depression).

## Methods

### Participants and procedures

Participants for the parent study, Operation: SAFETY (Soldiers and Families Excelling Through the Years) were USAR/NG soldiers and their partners recruited from 47 units throughout New York State between 2014 and 2015. Participants were recruited in person by project staff during drill weekends from USAR/NG units. During drill sessions, project staff consulted with soldiers about the study with a 10-min overview of the project and explained the overall goal of examining how the health and experiences of reserve soldiers and their partners influenced each other’s physical and mental well-being. Soldiers were informed that the study questionnaires would cover a broad range of general health topics, including physical and mental health, military service and deployment information, caffeine and substance use, sleep, romantic relationships, and social networks. All prospective participants were informed of the confidentiality procedures of the study, including being assured that their responses would not be shared with the military or their partner. Once soldiers were briefed, soldiers were invited to complete a brief screening procedure. All soldiers received an information packet to take home and discuss with their partner to determine interests.

To be eligible, the couple needed to be: (1) one member a current USAR/NG soldier, (2) married or living as if married, (3) soldier age between of 18 and 45 years old and parter age over 18, (4) speak and understand English, (5) able and willing to participate, and (6) report consuming at least one alcoholic beverage in the past year. If they met the eligibility requirements, participants were then asked to provide informed consent before completing five annual surveys. Surveys collected data on military, relationship, substance use, mental health, and additional variables. Participants were compensated $60 at baseline. This study was approved by the Army Human Research Protections Office, the Office of the Chief, Army Reserve, the Adjutant General of the National Guard, and University at Buffalo Institutional Review Board.

A total of 1074 couples were screened for Operation: SAFETY and 731 (68%) were eligible to participate. Of the eligible couples, 572 (78%) agreed to participate. Overall, 411 couples completed the baseline assessment. The current study examined data from the baseline assessment from the women (soldier or civilians) (*N* = 411)) in Operation: SAFETY. Among all women, the average age was 30.4 years (range 18–53) and the majority (85%) identified as non-Hispanic White. Additionally, almost all (90%) reported attending college (50% completing college or more) and 80% were currently employed (full or part-time). Approximately 12% of the sample reported a total household income of less than $19,999. Lastly, a majority (64%) of our sample was married and 25% of women reported being pregnant in the past year.

Among civilian and USAR/NG women, the average age was 30 years (range 18–53) and 81% and 86% identified as non-Hispanic White, respectively. In terms of educational attainment among civilian women, 48% completed college or more and 80% were currently employed (full or part-time). For women in USAR/NG roles, 40% completed college or more and 80% were currently employed (full or part-time). Approximately 12% of both samples reported a total household income of less than $19,999. There were slightly more married USAR/NG women compared to civilians (66% vs 63%, respectively). Finally, 31% of USAR/NG women reported being pregnant in the past year compared to 24% of civilian women (Table 1).

**Table 1:** Operation: SAFETY baseline sample characteristics

	Overall	Civilian	Military
	(N = 411)	(N = 320)	(N = 91)
	%(N) *M*(SD)		
Age	30.41 (6.73)	30.26 (6.90)	30.96 (6.12)
Race/Ethnicity			
Non-Hispanic White	84.91% (349)	85.94% (275)	81.32% (74)
Non-Hispanic Black	2.43% (10)	2.50% (8)	2.20% (2)
Hispanic	4.87% (20)	4.06% (13)	7.69% (7)
Other	5.60% (23)	5.31% (17)	6.59% (6)
Missing/Refused	2.19% (9)	2.19% (7)	2.20% (2)
Education			
> High School	1.46% (6)	1.88% (6)	0% (0)
High School	9.00% (37)	10.31% (33)	4.40% (4)
Some College	43.31% (178)	39.69% (127)	56.04% (51)
College+	46.23% (190)	48.13% (154)	39.56% (36)
Employment Status			
Full-time	59.61% (245)	60.94% (195)	54.95% (50)
Part-time	21.17% (87)	19.38% (62)	27.47% (25)
Not currently employed	19.22% (104)	19.69% (63)	17.58% (16)
Total Household Income			
> 19,999	11.68% (48)	11.56% (37)	12.09% (11)
$20,000–39,999	18.00% (74)	18.13% (58)	17.58% (16)
$40,000–59,999	16.55% (68)	15.00% (48)	21.98% (20)
$60,000–79,999	18.73% (77)	19.06% (61)	17.58% (16)
$80,000–99,999	11.19% (46)	11.25% (36)	10.99% (10)
$100,000–119,999	6.57% (27)	6.88% (22)	5.49% (5)
$120,000 +	7.79% (32)	7.19% (23)	9.89% (9)
Don’t Know	8.52% (35)	10.31% (33)	2.20% (2)
Refused	0.97% (39)	0.63% (2)	2.20% (2)
Relationship Status			
Married	63.50% (261)	62.81% (201)	65.93% (60)
Living with someone as if married	36.50% (150)	37.19% (119)	34.07% (31)
Past Year Pregnancy			
Yes	25.30% (104)	23.75% (76)	30.77% (28)
No	74.70% (307)	76.25% (244)	69.23% (63)

## Measures

### Alcohol use

Alcohol use was examined with the Alcohol Use Disorders Identification Test (AUDIT) (Babor et al., 2001; Saunders et al., 1993). The AUDIT is a 10-item measure that assesses participants’ current alcohol consumption, symptoms of dependence, and alcohol-related harm. Responses are rated on a 4-point Likert scale from “Never (0)” to “Daily or Almost Daily (3)”. Some example questions in this measure include: “How many drinks containing alcohol do you have on a typical day when you are drinking?” and “How often during the last year have you found that you were not able to stop drinking once you had started?” (Saunders et al., 1993). Total scores range from 0 to 40 and subscales range from 0 to 12 (dependence and consumption) and 0–15 (alcohol-related harm), with higher scores indicating greater problematic use (Saunders et al., 1993). This measure has been used in military populations previously (Crawford et al., 2013; Searle et al., 2015).

### Covariates

#### Mental health

Depression symptoms were assessed with the Patient Health Questionnaire (PHQ-8) (Kroenke et al., 2009), an 8-item measure assessing participants’ frequency of experiencing depression related symptoms within the past 2 weeks. This measure has been used in military samples previously (Smith et al., 2026). Responses for this measure are rated on a 4-point Likert scale from “Not at all (0)” to “Nearly every day (3)”. Example questions include: “little interest or pleasure in doing things,” “feeling tired or having little energy,” and “feeling down, depressed, or hopeless.” This analysis included the total depression score (range 0–24), where higher scores indicate greater depressive symptoms.

PTSD symptoms were measured using the PTSD-Checklist for DSM-5 (PCL-5) (Blevins et al., 2015; Weathers et al., 2013), a 20-item measure that indicates how much participants have been bothered by PTSD symptoms in the past month. Potential questions include being bothered by: “repeated, disturbing, and unwanted memories of the stressful experience” and “avoiding memories, thoughts, or feelings related to the stressful experience.” Responses are scored on a 5-point Likert scale from “Not at all (0)” to “Extremely (4)”. This analysis included the total PCL-5 score (range 0–80), where higher scores indicate greater PTSD symptomology. The PCL-5 has been used in military populations previously (Bovin et al., 2016; Searle et al., 2015; Wortmann et al., 2016), and total scores are efficient for assessing a wide range of subthreshold and clinical symptoms (Bovin et al., 2016).

### Participant characteristics

To determine motherhood status, women were asked “How many children do you currently have? Please include adopted or foster children and any stepchildren that live with you?” Women were dichotomized into groups of mothers (any value greater than zero) and non-mothers (value equal to zero). Specifically, 66% of women in the military had children compared to 59% of civilian women. We also assessed the mothers age (in years) as well as their current level of educational attainment (less than high school, high school, some college, college or more).

### Analytic plan

All statistical analyses were conducted using Stata version 15 (StataCorp, 2017). Descriptive statistics were used to summarize participant characteristics on key predictor and outcome variables. T-tests were used to assess differences in the continuous variables of age, depression, and PTSD. Additionally, a chi-square test was used to assess differences in the binary variable (education). Preliminary analyses were performed to determine differences in predictor and outcome variables based on participants’ motherhood status (Y/N), separated by military status (civilian vs USAR/NG soldier). Next, logistic and negative binomial regressions were used to examine alcohol use outcomes for civilian and military mothers separately. Logistic regressions were used to examine binary (AUDIT ≥ 8) variables while negative binomial regressions were used to account for the limited range of the examined count variables (AUDIT Total and subdomains) (Green, 2021). Adjusted models controlled for age, level of education, depression, and PTSD. Incidence rate ratios (IRR) or odds ratios (OR), 95% confidence intervals (CI), and significance levels are reported.

## Results

### Preliminary results

Preliminary analyses compared mothers and non-mothers separately on key demographic and mental health variables. Overall, 61% of the women in our sample had children. Among women with children, 24% (N = 60) were current USAR/NG soldiers and 76% (N = 190) were civilian mothers. The mean number of children women had at baseline was two (SD = 1.14), with the number of children ranging from one to 11. The age of the children in our sample ranged from one to 32 years, with 53% of the sample having adolescent children (18 years old or less).

There was a significant difference in the age of civilian and military mothers compared to non-mothers. Among both military and civilian women, women with children were significantly older than non-mothers ((Civilian mean age: 32.69 vs. 26.70 (*p* < 0.001); Military: mean age: 32.19 vs. 28.56 (*p* < 0.01), mother and non-mother, respectively)). There was no significant difference in educational attainment based on maternal status among USAR/NG soldiers. However, among civilian women, significant differences were identified, with mothers being less likely to obtain a college degree compared to non-mothers.

### Descriptive results

When assessing alcohol use outcomes among the total sample, the mean AUDIT total score was 3.54 (SD = 3.53, range = 0–29). Additionally, 9.98% (N = 41) of the sample reported an AUDIT score greater than 8 indicating potential problematic use. The mean consumption score was 2.66 (SD = 1.72, range = 0–9), the mean dependence score was .24 (SD = 1.02, range = 0–12), and the mean alcohol-related harm score was .65 (SD = 1.58, range = 0–12). In terms of mental health symptoms, the mean depression score was 4.61 (SD = 5.05, range = 0–24) and the mean PTSD score was 11.17 (SD = 13.70, range = 0–71). Among the total population of women in *Operation: SAFETY*, 61% were mothers of at least one child.

### Descriptive results by military status

Overall, 22.14% (N = 91) of women had ever served in a USAR/NG role. The two groups of women (civilian and military) in our sample had comparable AUDIT total scores, with a mean score of 3.55 (SD = 3.56, range = 0–29) for civilians and 3.49 (SD = 3.44, range = 0–24) for USAR/NG women. Additionally, consumption (mean_civ_ = 2.66, SD = 1.72 vs. mean_mil_ = 2.67, SD = 1.72; range 0–9), dependence (mean_civ_ = 0.25, SD = 1.09 vs. mean_mil_ = 0.20, SD = 0.72; range 0–12) and alcohol-related harm scores (mean_civ_ = 0.65, SD = 1.60 vs. mean_mil_ = 0.66, SD = 1.54, range 0–12) were comparable for civilian and military women, respectively. Mental health scores were comparable as well. The mean mental health scores for civilian women were as follows: depression (mean_civ_ = 4.53, SD = 5.08, range 0–24) and PTSD (mean_civ_ = 11.21, SD = 14.17, range 0–71). For military women, the mean mental health scores were as follows: depression (mean_mil_ = 4.87, SD = 4.98) and PTSD (mean_mil_ = 11.00, SD = 11.99) (Table 2).

**Table 2:** Demographic comparisons based on military and maternal status

	Civilian		USAR/NG	
	Mother	Non-Mother	Mother	Non-Mother
	(N = 190)	(N = 130)	(N = 60)	(N = 31)
	Mean (SD) or % (N)		Mean (SD) or % (N)	
Age	32.69 (7.07)	26.70 (4.79)***	32.19 (6.00)	28.56 (5.69)**
Education				
> High School	0.77% (1)	2.63% (5)***	0% (0)	0% (0)
High School	4.62% (6)	14.21% (27)	6.67% (4)	0% (0)
Some College	32.31% (42)	44.74% (85)	55.00% (33)	58.06% (18)
College+	62.31% (81)	38.42% (73)	38.33% (23)	41.94% (13)
Depression	4.83 (5.43)	4.10 (4.51)	5.08 (5.22)	4.45 (4.53)
PTSD	10.82 (14.14)	11.79 (14.25)	11.58 (11.64)	9.87 (12.76)

Significant results * = (*p* < 0.05), ** = (*p* < 0.01), *** = (*p* < 0.001)

### Motherhood status on alcohol use outcomes

#### Unadjusted civilian models (Table 3)

**Table 3:** Civilian alcohol use outcomes based on maternal status

	Unadjusted		Adjusted	
	Mother (N = 190)	Non-Mother (N = 130)	Mother (N = 190)	Non-Mother (N = 130)
	Mean or % (95% CI)		Mean or % (95% CI)	
AUDIT Total	2.68 (2.36–3.01)	4.82 (4.19–5.44)***	2.66 (2.33–3.00)	4.88 (4.19–5.57)***
AUDIT ≥ 8	4.73% (1.72–7.76)	19.23% (12.46–26.01)***	4.65% (1.59–7.72)	19.75% (11.61–27.89)**
Consumption	2.22 (2.01–2.44)	3.29 (2.98–3.60)***	2.18 (1.95–2.40)	3.40 (3.03–3.77)***
Dependence	0.14 (0.06–0.21)	0.42 (0.19–0.66)**	0.11 (0.38–0.17)	0.52 (0.17–0.87)**
Alcohol-Related Harm	0.34 (0.21–0.46)	1.10 (0.69–1.51)***	0.35 (0.20–0.50)	1.13 (0.63–1.63)***

Significant results * = (*p* < 0.05), ** = (*p* < 0.01), *** = (*p* < 0.001). Adjusted models controlled for mothers’ age, education, depression, and PTSD symptoms

Among civilian women, motherhood status was significantly associated with AUDIT total (IRR 0.56, 95% CI 0.47–0.67, *p* < 0.001) and AUDIT ≥ 8 (OR 0.21 95% CI 0.09–0.46, *p* < 0.001) scores. Motherhood status was also significantly associated with the subscales of consumption (IRR 0.67, 95% CI 0.59–0.77, *p* < 0.001), dependence (IRR 0.32, 95% CI 0.15–0.71, *p* < 0.01), and alcohol-related harm (IRR 0.31, 95% CI 0.18–0.25, *p* < 0.001). In all models, civilian mothers were less likely to report at risk alcohol use compared to non-mothers.

#### Adjusted civilian models

When adjusting for age, education, depression, and PTSD, all previous models remained significant. Results for the adjusted regressions were as follows: AUDIT total (IRR 0.55, 95% CI 0.44–0.67, *p* < 0.001), AUDIT ≥ 8 (OR 0.18, 95% CI 0.07–0.49, *p* < 0.01), consumption (IRR 0.64, 95% CI 0.54–0.75, *p* < 0.001), dependence (IRR 0.20, 95% CI 0.08–0.52, *p* < 0.01), and alcohol-related harm (IRR 0.31, 95% CI 0.16–0.58, *p* < 0.001). In adjusted models, women’s depression score was a significant covariate in the association between motherhood status and AUDIT total (IRR 1.04, 95% CI 1.01–1.07, *p* < 0.01), consumption (IRR 1.02, 95% CI 1.01–1.04, *p* < 0.05), and dependence (IRR 1.13, 95% CI 1.03–1.24, *p* < 0.05). Specifically, higher depression scores were significantly associated with increased negative alcohol use outcomes. The mothers age, level of education, and PTSD symptoms were not significant covariates in the relationship between motherhood status and any alcohol use outcome (ps > 0.05).

#### Unadjusted military models (Table 4)

**Table 4:** USAR/NG alcohol use outcomes based on maternal status

	Unadjusted		Adjusted	
	Mother (N = 60)	Non-Mother (N = 31)	Mother (N = 60)	Non-Mother (N = 31)
	Mean or % (95% CI)		Mean or % (95% CI)	
AUDIT Total	3.28 (2.60–3.97)	3.90 (2.81–4.99)	3.50 (2.78–4.21)	3.47 (2.55–4.40)
AUDIT ≥ 8	5.00% (0–10.51)	12.90% (1.10–24.70)	5.20% (0–10.82)	12.55% (0.75–24.34)
Consumption	2.56 (2.14–2.98)	2.87 (2.25–3.49)	2.67 (2.23–3.11)	2.66 (2.08–3.23)
Dependence	0.13 (0.02–0.25)	0.32 (0.02–0.63)	0.17 (0.01–0.33)	0.20 (0.02–0.39)
Alcohol-Related Harm	.63 (.24–1.02)	0.71 (0.11–1.31)	0.72 (0.24–1.19)	0.55 (0.08–1.02)

Significant results * = (*p* < 0.05), ** = (*p* < 0.01), *** = (*p* < 0.001). Adjusted models controlled for mothers’ age, education, depression, and PTSD symptoms

Among women in USAR/NG roles, motherhood status was not significantly associated with participants’ AUDIT total, AUDIT ≥ 8, consumption, dependence, or alcohol-related harm scores.

#### Adjusted military models

After adjusting for age, level of education, depression, and PTSD; no significant results emerged for any alcohol use outcomes measured. However, USAR/NG women’s PTSD score was significantly associated with their AUDIT total (IRR 1.02, 95% CI 1.01–1.04, *p* < 0.05) and consumption scores (IRR 1.02, 95% CI 1.01–1.03, *p* < 0.05), with higher PTSD scores being associated with worse alcohol use outcomes. Additionally, education was significantly associated with consumption (IRR 1.45, 95% CI 1.13–1.84, *p* < 0.01) and alcohol-related harm (IRR 2.96, 95% CI 1.14–7.69, *p* < 0.05), where greater levels of education were associated with higher consumption and alcohol-related harm scores.

## Discussion

The present study is among the first to examine the effects of motherhood status on the alcohol use outcomes of a sample of USAR/NG soldier and civilian women. Overall, 10% of the women in our sample reported potentially harmful alcohol use scores (indicated by AUDIT total scores greater than or equal to 8). Importantly, the civilian and USAR/NG women in our sample were comparable across demographic, substance use, and mental health variables. By identifying whether motherhood serves as a protective factor for problematic alcohol use among women in USAR/NG roles, this study has the potential to inform future prevention strategies and support interventions that can improve maternal and military outcomes such as soldier readiness and retention.

Using the Occupational Mental Health Model as a foundation, this research helps to establish how personal demands of motherhood and occupational demands related to being an USAR/NG solder (traditional and high risk) can combine to influence outcomes and goals (Adler & Castro, 2013). Consistent with our hypothesis, motherhood was significantly associated with lower risk alcohol use outcomes among civilian women. Compared to non-mothers, civilian mothers reported lower AUDIT total, AUDIT ≥ 8, and subscale scores. These findings support the idea that motherhood can serve as a protective factor against alcohol use problems in civilian populations. However, this protective effect of motherhood was not observed among women in USAR/NG roles. These findings may suggest that the protective effects typically associated with motherhood may diminish for mothers with multiple competing complex roles. This may be particularly relevant for servicewomen that experience unique sex-specific social and occupational contexts that can influence health outcomes.

### Motherhood and alcohol use among civilian women

Our findings among civilian women are supported by previous research indicating that motherhood is associated with decreased alcohol use problems among women in the general population (Balan et al., 2014; Patsouras et al., 2025). In civilian contexts, social norms and caregiving responsibilities can create barriers that ultimately discourage maternal alcohol use (Fleming et al., 2025). Our adjusted results further demonstrate that this protective effect remained even after accounting for women’s age, education, and mental health scores. It is important to note however, that research on drinking behaviors of all mothers has received very limited attention, potentially due to stigma (Fleming et al., 2025).

### The influence of complex competing roles on alcohol use

Working mothers take on multiple competing complex roles related to balancing work, family, and social responsibilities which may negatively impact health outcomes. Our findings support this idea and suggest that occupational context is extremely important in shaping ones alcohol use behaviors. Women in military roles experience unique sex-specific exposures due to their service that may alter alcohol use perceptions and ultimately limit the protective effects typically associated with motherhood. Compared to men, servicewomen are more susceptible to negative physical, psychological, and social outcomes which may confound with competing complex roles such as motherhood to produce negative health outcomes, including problematic alcohol use.

As service members, civilians, and mothers, USAR/NG soldiers occupy unique roles, often resulting in them reporting feeling as though they manage a dual identity of mother versus servicewomen (Williamson et al., 2023). Due to childcare concerns, occupational challenges, and gender stereotypes, servicewomen have been found to report more parental role conflict (Williamson et al., 2023). According to prior studies in military samples, service provides a unique set of challenges for military mothers such as maternal separation anxiety (Barnes et al., 2016). Due to the part time structure of USAR/NG service, frequent transitions can disrupt the social responsibilities of soldiers, especially women. Military experiences such as extended time away from home due to trainings or deployments, in combination with responsibilities on the home front may interact to uniquely impact the alcohol use outcomes of servicewomen (Henion et al., 2024; Hoopsick et al., 2018; Mathes Winnicki et al., 2024).

### Implications and future directions

This study contributes to the literature on two very important and underdeveloped research areas, including maternal alcohol use and military women’s health. The topic of maternal alcohol use among all women is a severely underdeveloped area of research. For USAR/NG women specifically, more research is needed to better understand and address the unique stressors associated with multiple intersecting identities (military, civilian, and mother) and their impact on alcohol use behaviors. For example, focusing on sex-based concerns surrounding deployment related stress, caregiving challenges, and access to mental health services could positively influence alcohol related outcomes among this population. As stated by Adler and Castro (2013), the Occupational Mental Health Model and considering occupational context is important for determining necessary health resources, appropriate monitoring efforts, and potential interventions that can promote an organizations systematic approach to caring for its members to prevent, reduce, and treat mental health problems encountered by members.

Drinking during motherhood is complex and will likely involve changing cultural norms around women’s alcohol use (Fleming et al., 2025). Due to the projected increase of women in military and veteran roles, strategies should be implemented to better support this demographic. First, additional research is needed to better understand the relationship between alcohol use and motherhood among women in the USAR/NG and other military populations. Future research should assess how subsequent factors such as stress, social support, marital quality, and deployment experiences impact the alcohol use of USAR/NG women. Additionally, as stated previously, there is limited research on alcohol use of women across the entire life course. Future work should aim to understand how intersecting roles such as motherhood and military service influence alcohol use patterns and outcomes among women over time. Lastly, our use of a cut score of 8 or higher may be a conservative estimate of hazardous alcohol use and future studies should also include an AUDIT cut point of 7, which may be more appropriate for mapping DSM-5 criteria for AUD (Ingesson-Hammarberg et al., 2024).

In addition to research, this study has various implications for both clinical practice and military policy. On the individual level, increased attention to education on alcohol associated harms, mental health disorders, and family readiness programs, especially for women in military roles that are mothers, could decrease the risk of adverse health outcomes in this population. Other personal resources may include coping strategies, including problem-focused coping or active coping (Adler & Castro, 2013). Additionally, practices can be implemented to improve the experiences and outcomes of women in the military by providing support for additional female healthcare providers, training on the unique needs of servicemembers that are women, and by developing sex-specific screening tools and interventions that aim to reduce alcohol misuse (Adams et al., 2021).

### Limitations

The current study is subject to limitations. First, our sample is entirely from USAR/NG units across New York state, and all participants had to be in couples at baseline. Although this may limit the generalizability of our findings, our sample’s characteristics are consistent with reservist nationally and more than half of all service members are married. Additionally, there were no single mothers in our sample at baseline. Future research is needed to assess how single motherhood influences role responsibilities and alcohol use outcomes among military women. As all of the women in our study are associated with military families in some way, it would also be useful to recruit a sample of women with no military associations. Next, this analysis was cross sectional. While this hinders our ability to claim causal inference, future longitudinal research can help to assess the alcohol use of mothers in military roles over time. Lastly, the data for this research is self-reported. Despite this limitation, self-report data is the standard for behavioral research and established measures were used.

### Strengths

Despite the previously stated limitations, this research has many strengths and focuses on various understudied populations. Our sample contains women in USAR/NG roles. As stated, previously, USAR/NG soldiers have unique identities despite sharing similar experiences to their active-duty counterparts. Further, this research focuses on military women who are mothers, an important and under-represented group given recent trends in military demographics. While focusing on USAR/NG mothers is a strength alone, using a comparison group of civilian mothers and non-mothers supports the validity of our findings. Not only does this research enhance the literature on working mothers, it also adds to the understanding of sex-based alcohol use outcomes in military populations and highlights the importance of considering the impact of family and caregiver influences on military outcomes.

## Conclusion

This study enhances the current literature on the health of women in military roles by highlighting the influence of motherhood on alcohol use outcomes. Our findings indicate that the protective effects of motherhood on alcohol use typically found among civilian women, may not be observed by women with multiple complex and competing roles such as USAR/NG servicewomen. While civilian mothers were significantly less likely to report problematic alcohol use, this effect was not found among women in USAR/NG roles. These results emphasize the importance of examining the influence of social and occupational factors on the alcohol use of women. The complex relationship between motherhood and military service is important for current and future military personnel and underscore the need for developing interventions that promote the health and well-being of military mothers and their families.

## Data Availability

Not applicable.
